# DGPT news: Rudolf Buchheim Award 2024

**DOI:** 10.1007/s00210-025-04114-x

**Published:** 2025-04-11

**Authors:** Rebekka Lambrecht

**Affiliations:** 1 Biochemical Pharmacology, Department of Biology, University of Konstanz, Universitaetsstrasse 10, 78464 Konstanz, Germany; 2 Department of Systems Immunology, Weizmann Institute of Science, Herzl St. 234, 7610001 Rehovot, Israel

**Keywords:** Paracetamol, Drug-induced liver injury (DILI), Cell death, Oxidative stress, Mitochondria, Energy metabolism

## Abstract

The Rudolf Buchheim Award is an annual prize awarded by the German Society for Experimental and Clinical Pharmacology and Toxicology (DGPT) to recognize outstanding work from young researchers in the fields of pharmacology and toxicology. The 2025 Rudolf Buchheim Award recognizes advancements in understanding drug-induced liver injury. This year’s recipient, Rebekka Lambrecht, investigated acetaminophen (APAP)-induced liver toxicity in the lab of Prof. Thomas Brunner at the University of Konstanz. Her research highlights the role of mitochondrial damage and oxidative stress in driving hepatocyte death, revealing that metabolic reprogramming can enhance cell survival and demonstrating the mechanism APAP toxicity inhibits apoptosis and shifts cells toward necrosis. Her findings provide a deeper understanding of APAP hepatotoxicity and suggest potential therapeutic strategies aimed at stabilizing mitochondrial function and mitigating oxidative stress in drug-induced liver injury.

## Short communication

Drug-induced liver injury (DILI) remains a major clinical concern, with paracetamol (acetaminophen, APAP) overdose being the leading cause of acute liver failure in Western countries (Stravitz and Lee 2019). Despite its widespread use as a pain reliever, excessive doses of paracetamol can overwhelm the liver’s detoxification capacity, leading to irreversible liver damage, which ends often fatal if left untreated. While treatment options, such as N-acetylcysteine, exist, their effectiveness is limited to a narrow time window, underscoring the urgent need to better understand the cellular processes driving APAP-induced hepatotoxicity.

This year’s Rudolf Buchheim Award from the DGPT was received by Rebekka Lambrecht and her research in the lab of Prof. Thomas Brunner, University of Konstanz, elucidating cellular mechanisms underlying APAP-induced liver injury. Her work focuses on mitochondrial damage and liver cell death and was published in *Cell Death & Differentiation* and *Cell Death & Disease* (Lambrecht et al. 2023, 2024).

**Fig. 1:**
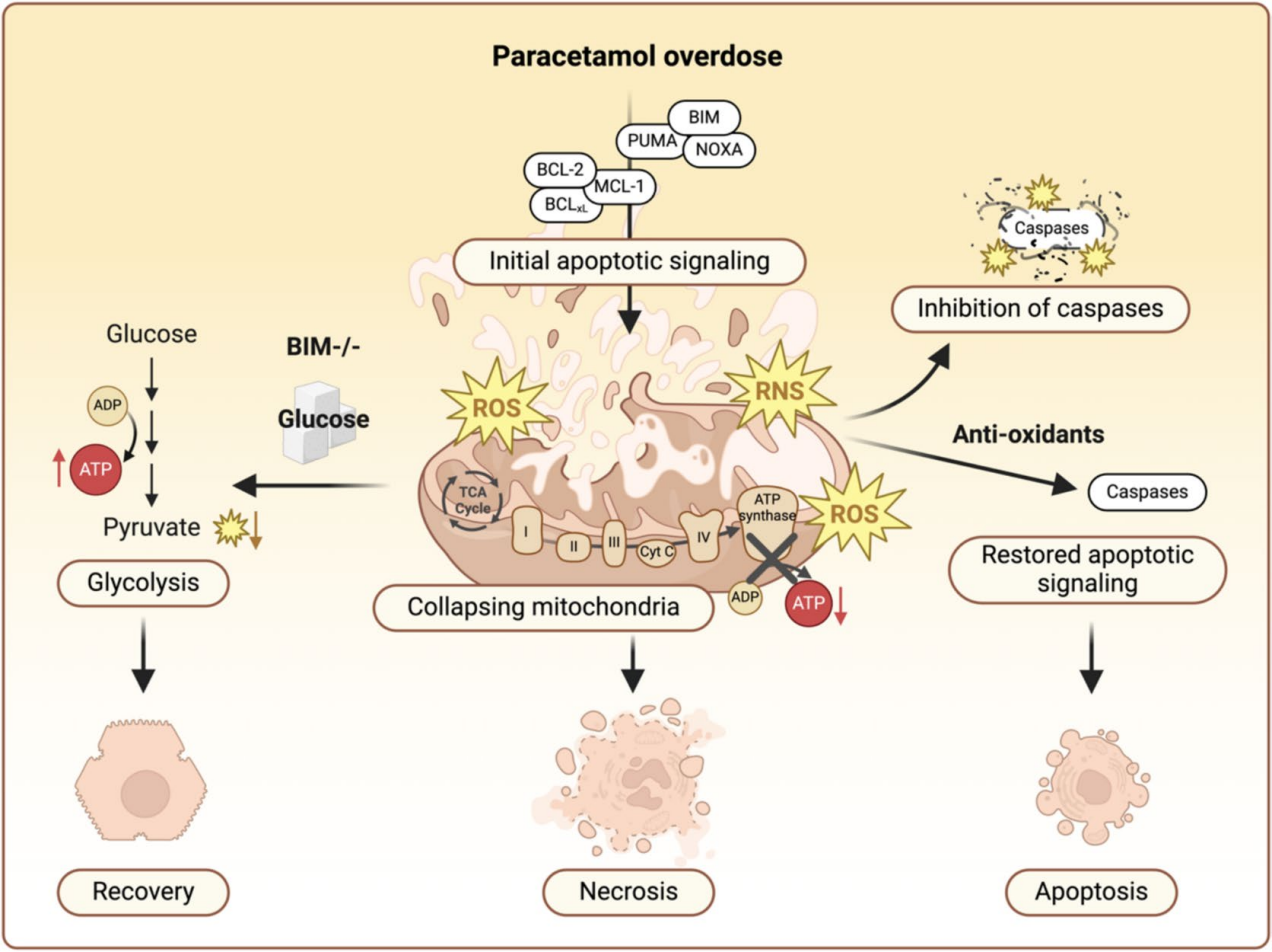
Balancing act: how energy metabolism and oxidative stress determine hepatocyte death in paracetamol overdose. Paracetamol (APAP) overdose triggers mitochondrial damage in hepatocytes, initiating apoptotic signaling, while impairing ATP production and inducing excessive oxidative stress. This results in a self-amplifying loop of mitochondrial injury, ultimately leading to mitochondrial collapse and hepatocyte necrosis. Shifting cellular energy production towards glycolysis, either through glucose supplementation or BIM deficiency, reduces mitochondrial dependency, alleviating oxidative stress and energy shortage, thereby allowing hepatocytes to recover from APAP intoxication. Additionally, counteracting APAP-induced oxidative stress with antioxidants restores caspase activity and facilitates the execution of apoptosis, offering an alternative, less inflammatory cell death pathway

APAP overdose has long puzzled researchers with its mixed cell death signals (Jaeschke et al. 2019)—it triggers early apoptotic events in liver cells but ultimately results in necrotic cell death. Cell death is commonly categorized into apoptosis, a programmed cellular process with limited tissue impact, and necrosis, an uncontrolled, lytic and often harmful form of cell death, and other forms of programmed cell death that share features with necrosis (Yuan and Ofengeim 2023). APAP is known to disrupt mitochondrial integrity, causing a catastrophic energy crisis and unleashing excessive reactive oxygen and nitrogen species (ROS, RNS) in liver cells (Ramachandran and Jaeschke 2023). As mitochondria collapse, hepatocytes lose their ability to sustain vital functions, culminating in necrosis that amplifies tissue damage and can lead to fulminant acute liver failure (Fig. 1).

Lambrecht’s first study uncovered a non-canonical role for the apoptosis regulator BIM in shaping liver cell fate during APAP overdose (Lambrecht et al. 2023). She demonstrated that beyond its classical pro-apoptotic role, BIM also regulates energy metabolism and mitochondrial function. Deleting BIM shifts liver cell metabolism from mitochondrial oxidative phosphorylation towards glycolysis. This metabolic reprogramming not only boosts overall ATP production but also lowers ROS levels, allowing cells to better withstand mitochondrial damage. Importantly, hepatocytes relying on glycolysis are significantly more resilient to APAP toxicity, while those dependent on oxidative phosphorylation suffered severe damage. Supporting experiments, including glucose modulation and deletion of mitochondrial fusion proteins, reinforced the centrality of energy metabolism in determining hepatocyte survival during drug toxicity. Remarkably, even direct glucose administration, enhancing glycolysis, conferred protection in both hepatocytes and mouse models (Fig. 1), pointing to potential therapeutic strategies for APAP-induced liver injury.

In her second study, Lambrecht addressed the longstanding paradox of initial apoptotic signaling followed by necrosis during APAP intoxication (Lambrecht et al. 2024). While ATP depletion from impaired mitochondrial respiration had been a suspected culprit, her findings instead identified oxidative stress as the key driver for this cell fate decision. APAP not only fails to activate caspases, the enzymes central to apoptosis, but actively inhibits their activation, even in the presence of classical apoptotic triggers. The research suggests that excessive ROS levels generated during APAP overdose chemically modify caspases, preventing their activation, halting the apoptotic process, and shifting cells towards necrosis. Importantly, the researchers found that antioxidant treatment restored caspase activity, nudging the cells back towards apoptosis (Fig. 1). This highlights the critical role of the cellular redox state in governing cell death decisions.

By dissecting the complex interplay between mitochondrial function, energy metabolism, and cell death pathways, Lambrecht’s research advances our understanding of APAP-induced liver failure and identifies novel therapeutic targets for mitochondria- and oxidative stress-related intoxications. Approaches aimed at stabilizing mitochondrial function or enhancing glycolysis could potentially mitigate liver damage in APAP overdose patients, extending the window for effective treatment.

## Data Availability

There were no data used for this article.
